# A nosological approach to brief psychotic disorders and acute and transient psychoses

**DOI:** 10.1192/j.eurpsy.2024.320

**Published:** 2024-08-27

**Authors:** C. M. Coelho

**Affiliations:** Psychiatry, Centro Hospitalar e Universitário de Coimbra, Coimbra, Portugal

## Abstract

**Introduction:**

Acute and transient psychoses (International Classification of Diseases) and Brief Psychotic Disorders (Diagnostic and Statistical Manual of Mental Disorders) constitute heterogeneous nosological groups, which have undergone successive reformulations in the past decades, remaining doubts regarding their diagnostic validity and independence.

**Objectives:**

This work aims to review the nosological evolution of these complex and neglected groups.

**Methods:**

A review of the literature was conducted using PubMed and The Cochrane Library. The following terms were used: “acute and transient psychoses”; “brief psychotic disorders”; “cycloid psychosis”; “reactive psychosis”.

**Results:**

Since the early 20th century, a group of non-affective psychoses with acute onset and brief duration have been described in different countries and under various names, such as bouffeé delirante, reactive psychosis or cycloid psychosis, denominations still present in ICD-9. In presente-day classifications, as ICD-10 and DSM-IV, an effort was made to homogenise the various regional and national concepts creating the group of ‘Brief Psychoses’ (DSM) or ‘Acute and Transient Psychotic Disorders’ (ICD). The marked heterogeneity and low diagnostic stability of these groups, mainly based on temporal criteria, has posed significant obstacles to further research and conceptualization. Given these difficulties, the latest revision of the International Classification of Diseases (ICD-11) brought about a substantial change, restricting this diagnosis to polymorphic psychotic conditions of acute onset and rapid resolution, subgroup with greater diagnostic stability and characteristics distinct clinical features.

**Conclusions:**

The relevance of a better clarification for this nosological group is evident in the successive changes over the last century. ICD 11, once again, substantially changed the diagnostic criteria and the scope of this nosological entity, leaving doubts about the independent nature of this group, its connection to schizophrenia, as an attenuated form (more common in women and in developing countries), or even as a form of psychosis that is closer to affective disorders (due to its clinical evolution). Although little explored, this issue remains a source of doubt and interest, calling into question the Kraepelinian dichotomy for the so-called endogenous psychoses.

**Disclosure of Interest:**

None Declared

